# Mechanisms of neuro-robotic prosthesis operation in leg amputees

**DOI:** 10.1126/sciadv.abd8354

**Published:** 2021-04-21

**Authors:** Giacomo Valle, Albulena Saliji, Ezra Fogle, Andrea Cimolato, Francesco M. Petrini, Stanisa Raspopovic

**Affiliations:** 1 Laboratory for Neuroengineering, Department of Health Sciences and Technology, Institute for Robotics and Intelligent Systems, ETH Zürich, 8092 Zürich, Switzerland.; 2 SensArs Neuroprosthetics, Saint-Sulpice CH-1025, Switzerland.

## Abstract

Above-knee amputees suffer the lack of sensory information, even while using most advanced prostheses. Restoring intraneural sensory feedback results in functional and cognitive benefits. It is unknown how this artificial feedback, restored through a neuro-robotic leg, influences users’ sensorimotor strategies and its implications for future wearable robotics. To unveil these mechanisms, we measured gait markers of a sensorized neuroprosthesis in two leg amputees during motor tasks of different difficulty. Novel sensorimotor strategies were intuitively promoted, allowing for a higher walking speed in both tasks. We objectively quantified the augmented prosthesis’ confidence and observed the reshaping of the legs’ kinematics toward a more physiological gait. In a possible scenario of a leg amputee driving a conventional car, we showed a finer pressure estimation from the prosthesis. Users exploited different features of the neural stimulation during tasks, suggesting that a simple prosthesis sensorization could be effective for future neuro-robotic prostheses.

## Teaser

Restoring neural sensory feedback to amputees improves the exploitation of leg prosthesis during motor tasks.

## INTRODUCTION

Commercially available lower-limb prostheses do not provide voluntary active control nor sensory feedback to the user (*1*). Consequently, amputees using these systems often complain about the need to rely on visual cues during everyday prosthesis use (*2*, *3*). More recently, research groups (*4*–*6*) and prosthetic companies (*7*) have proposed devices that provide the users with active control of the prosthesis. However, there are no commercially available leg prostheses that provide sensory feedback to the users (i.e., real-time information about the movement of the prosthesis itself or about the interaction with the ground). Sensory feedback provided by foot sole mechanoreceptors, leg muscle spindles, and tendon organs is crucial for controlling balance and movement in humans (*8*–*11*). From the perspective of neural control and biomechanics, the control of gait requires kinematic and dynamic coordination of the limbs and muscles, multisensory fusion, and robust control mechanisms. Sensory feedback from muscle and skin afferents, as well as other sensory modalities, dynamically influences adapting of the pattern of locomotion to the requirements of the environment (*12*).

Because of the lack of feedback, users do not perceive the prosthesis as part of their own body (i.e., low embodiment) (*13*, *14*), which increases the cognitive effort when using the device itself, affecting its acceptability (*14*–*16*), and they experience dangerous falls (*17*). These facts cause a confidence reduction of the subject in the prosthesis use (i.e., they are afraid to fall if relying on it), resulting in 60% of lower-limb amputees abandoning the prosthesis (*18*–*20*). Because of the lack of confidence, amputees produce counterbalancing movements that increase fatigue (*21*). The resulting abnormal kinematics and postural asymmetries produce augmented metabolic cost, then fatigue, and occasionally heart failures (*21*).

To solve this issue, different techniques were presented to provide sensory feedback to the prosthesis users exploiting noninvasive stimulation (*22*–*24*), surgical approaches (*6*), or direct nerve stimulation (*25*–*29*). In particular, the neurostimulation is able to restore quasi-somatotopic, quasi-homologous, and intuitive sensory feedback to the user that combines tactile and position information (*26*). Recently, it has been shown that real-time intraneural feedback in a leg prosthesis improves mobility in above-knee (transfemoral) amputees (*25*, *26*). Although there is evidence that changing the sensory feedback leads to kinematic, kinetic, and neuromuscular adaptations in static as well as dynamic conditions (*30*), the precise effects of artificial sensory feedback, integrated in a prosthetic leg, on users’ sensorimotor strategies and its implications for future wearable robotics devices development are not investigated yet. Moreover, an objective quantification of user prosthesis confidence is still missing [i.e., subjects were asked to provide personal reports on experienced confidence (*25*)].

The purpose of this work is to unveil how artificial neural feedback implemented in a sensorized bionic leg triggers novel sensorimotor strategies and augments confidence during walking in lower-limb amputees. As a consequence, we also suggest the characteristics of a sensorized robotic device to be considered of great importance in the design of future prostheses. To do so, we assessed the gait of two transfemoral amputees implanted with transversal intraneural multichannel electrodes (TIMEs) (*31*) in their tibial nerves to elicit tactile and movement sensations integrated in a sensorized leg prosthesis (*25*, *26*). In particular, we measured mobility, self-confidence, spatiotemporal, force, and kinematics parameters during gait while the subjects were performing two possible motor tasks of everyday life. Velocity was the selected feature to asses mobility, being one of the most relevant and straightforward indicators in clinical practice (*32*). They were asked to perform an easy task—walking over ground (OT)—and a challenging task—ascending and descending stairs (ST). All the gait parameters were collected with and without neuroprosthetic intervention. Last, we tested with one subject whether sensory feedback could improve fine pressure exerted with the prosthetic leg when using a car accelerator pedal. Leg amputees suffer an impairment in prosthetic force control capabilities not only during locomotion (*33*) but also during stationary force generation (*34*, *35*).

This study reports how personalized neuroprostheses (*36*) providing rich and multimodal sensory information in real time, while enhancing amputees’ mobility (*37*) and prosthesis’ confidence in ecological conditions (*38*), could modify the biomechanics of gait and improve fine force control.

## RESULTS

**Fig. 1:**
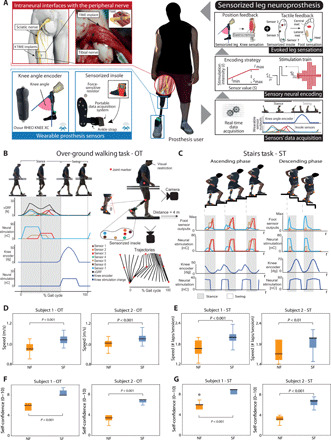
Nerves-connected leg prostheses enhance mobility during OT and ST. (**A**). Schematic representation of the sensorized prosthetic leg and its main components. The involved subjects were transfemoral amputees equipped with (i) neural interfaces in their tibial nerve, (ii) RHEO KNEE XC with an angle knee encoder, (iii) sensorized insoles, (iv) microprocessor programmed for a real-time acquisition and conversion of the sensors’ readouts to neural stimulation, and (v) a portable neurostimulator. Real-time tactile sensory feedback was provided using three electrode channels eliciting sensations in three positions under the phantom foot associated with three sensors of the insoles. Real-time position feedback, related to the prosthetic knee flexion/extension, was also provided using an electrode channel evoking a sensation referred to the phantom calf muscle. (**B**) OT. A subject while walking over ground, synchronous sensorized insole and knee encoder readouts, and encoded currents injected into the TIMEs. (**C**) ST. A subject while climbing (left) and descending stairs (right). Synchronous sensorized insole and knee encoder readouts, and encoded currents injected into the TIMEs. In both tasks, markers were placed on the main leg joints (Materials and Methods) for the kinematics gait analysis. (**D**) Box plot of the gait speed (meter per second) with (SF) and without (NF) neural sensory feedback during OT for subjects 1 and 2. *n* = 30 sessions per condition. (**E**) Box plot of the gait speed (number of laps per session) with (SF) and without (NF) neural sensory feedback during ST for subjects 1 and 2. *n* = 24 sessions per condition. Box plots of the self-reported confidence (**F**) during OT and (**G**) ST for subjects 1 and 2 in NF and SF are displayed. *P* values of the Wilcoxon test are shown. (Photo credit: Giacomo Valle, ETH Zurich).

The participants (subjects 1 and 2) (table S1) had suffered a transfemoral amputation, because of traumatic events. They received implants of four transversal intraneural electrodes (*31*) in the distal section of the tibial nerve (Fig. 1A and fig. S1). The neural sensory feedback was characterized and restored through a sensorized bionic leg [as described in (*26*)]. The bionic leg was equipped with pressure sensors under the foot and encoder in the knee. The neural stimulation was driven (through encoding algorithms; Materials and Methods and Fig. 1A and fig. S1) by the readout of these sensors. As a result, this neural stimulation provided the subjects with the continuous tactile and position information of the prosthetic leg over the entire gait cycle. The participants were not naïve to the neural stimulation, because they were part of a 3-month clinical trial, in which the first month was completely dedicated to the mapping procedure (*25*, *26*).

**Fig. 2:**
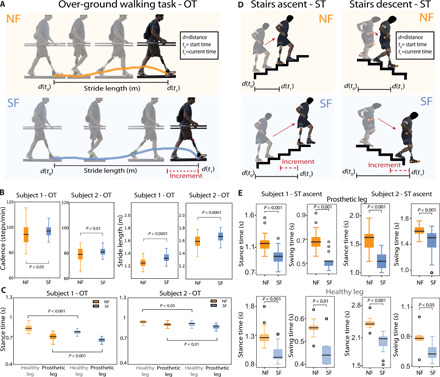
Sensory feedback activates novel motor strategies. (**A** and **B**) The length of the stride during OT is displayed for subject 1 (*n* = 45) and subject 2 (*n* = 50). Cadence during OT is reported for subject 1 (*n* = 100) and subject 2 (*n* = 110). (**C**) Stance time during OT is reported for both legs (healthy and prosthetic) in both subjects. *n* = 46 for subject 1 and *n* = 30 for subject 2 for condition and leg. (**D** and **E**) Stance time and swing time during the ascending phase of the ST are shown for both subjects and legs in NF and SF conditions. For the prosthetic leg, *n* = 24 for both subjects while for the healthy leg *n* = 22. All data are shown as box plots with (SF) and without (NF) neural sensory feedback during OT and ST. *P* values of the Wilcoxon test are shown. (Photo credit: Giacomo Valle, ETH Zurich).

To study the effects of the intervention, we designed two possible motor tasks of everyday life, one among the easiest and other among the most challenging for amputees: The subjects were asked to walk over ground (OT) with visual restriction (Figs. 1B and 2A) or to ascend and descend stairs (ST) (Figs. 1C and 2D). During these tasks, we measured ambulation speed, spatial and temporal features of gait (e.g., cadence, stride length, and stance/swing time), weight distribution on both legs, and kinematics of the main leg joints. The data were acquired using sensorized insoles, current controller storage, a knee encoder, joint markers, and cameras. These two tests were conducted in two different conditions: with (SF) and without (NF) neural sensory feedback.

During the OT, the subjects were asked to walk on a flat runway at their comfortable pace. Both subjects had greater mobility when sensory feedback was provided (*P* < 0.001) (Fig. 1D). This was measured by their gait speed. In subject 1, we observed an increase of around 10 cm/s in the SF condition as compared to the NF condition, which corresponds to an increase in speed of approximately 10%. In subject 2, we measured an increase of around 5 cm/s, this being an improvement of around 5%. In this task, the subjects were visually restricted to assess the sole impact of sensory feedback on the prosthesis avoiding the continuous inspection of the prosthesis while walking (action that they are used to do in everyday life). The subjects were also asked to walk on an angular staircase in sessions of 30 s, during the ST [ascending and descending stairs are among the most challenging situations in an amputee’s daily life (*39*)]. The mobility (number of laps) achieved by the two subjects with the SF was higher than the mobility without stimulation NF (*P* < 0.01) (Fig. 1E). These tests were more extensive in number of sessions and spanning over several days of tests, with respect to the previously reported data (*26*). In particular, subject 1 improved his speed (laps per session) from 1.71 ± 0.23 to 1.91 ± 0.24 and subject 2 from 1.67 ± 0.25 to 2.05 ± 0.11.

In addition, after every session of motor task, we asked the subjects to rate their self-confidence (from 0, no confident at all, to 10, extremely confident) while performing the tasks with and without sensory feedback. The results indicate that the subjects have higher self-confidence in both OT and ST (*P* < 0.001, Wilcoxon test; Fig. 1, F and G) when the intraneural sensory feedback (SF) was provided compared to NF. The subject 1 confidence passed from 5.9 ± 0.5 to 8.4 ± 0.4 in OT and from 5.8 ± 0.8 to 8.7 ± 0.4 in ST. In subject 2, the self-confidence passed from 3.4 ± 0.5 to 6.7 ± 0.4 in OT and from 3.3 ± 0.5 to 6.4 ± 0.7 in ST.

In the two tasks, the motor strategies of the gait were different between SF and NF conditions in both subjects. During the OT, both patients had a longer stride length in the SF condition compared to without sensory feedback (*P* < 0.001) (Fig. 2B and movie S1). In both subjects, the increase was of around 7 cm (Fig. 2B). A higher stride is a sign of higher gait confidence (*40*). We also observed that the cadence of subject 1 was three steps per minute significantly higher in the SF condition compared to without feedback, this corresponding to a growth of about 4% (*P* < 0.05) (Fig. 2B). In subject 2, we also saw a higher cadence in the SF condition with a significant increase of about two steps per minute (*P* < 0.001). At the same time, with sensory feedback, we observed a significant reduction (8% for subject 1 and 5% for subject 2) in the stance time of both the prosthetic and healthy legs in both subjects (*P* < 0.05) (Fig. 2E). On the other hand, no significant difference in swing time between the feedback conditions in either subjects were found (fig. S2A). The stance ratio remained constant (60% for subjects 1 and 2) on both legs (fig. S2B). In subject 1, when the sensory feedback was provided, stance and swing time were not different between healthy and prosthetic legs (fig. S3). During the ST, we found that in all cases, stance and swing time were significantly reduced when the subjects were provided with the neural feedback compared to the NF condition (*P* < 0.05) (Fig. 2E). During the ascending phase on the prosthetic side, the stance time reduction was of 20% in subject 1 and of 25% in subject 2, and the swing time reduction was of 25% in subject 1 and of 21% for subject 2. At the same time, the amounts of reduction obtained for the healthy leg were 25% for subject 1 and 18% for subject 2 in the stance time and 18% for subject 1 and 19% for subject 2 in the swing time (Fig. 2E). During the descending phase of ST on the prosthetic side, the stance time reduction was of 16% in subject 1 and of 24% in subject 2, and the swing time reduction was of 4% in subject 1 and of 12% for subject 2. For the healthy side, the stance time reduction was of 13% for subject 1 and 16% for subject 2, and the swing time reduction was of 18% for subject 1 and 23% for subject 2 (fig. S4).

**Fig. 3:**
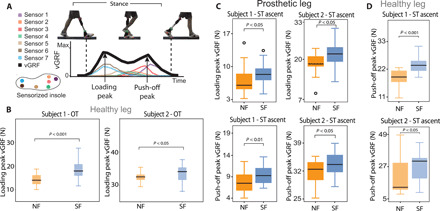
Quantification of the augmented prosthesis confidence. (**A**) The vGRF was calculated on both legs using the pressure sensors of the sensorized insoles. The loading peaks and the push-off peaks were measured on both legs. (**B**) The loading peaks measured on the healthy side showed a difference between SF and NF in both participants. *n* = 48 per subject and per condition. (**C**) Loading peaks and push-off peaks measured during the ascending phase in ST on the prosthetic side in both subjects are shown. *n* = 24 per subject and per condition. (**D**) Push-off peaks measured on the healthy side during the ascending phase in ST in both subjects are displayed. *n* = 16 per subject and per condition. All data are shown as box plots for neural sensory feedback (SF) and for without (NF) during OT and ST. *P* values of the Wilcoxon test are shown. (Photo credit: Stanisa Raspopovic, ETH Zurich).

Analyzing the vertical ground reaction forces (vGRFs) from the sensorized insoles during the two motor tasks, we extracted the loading peak and the push-off peak on both legs (Fig. 3A and table S2). In the OT, the loading force peak, measured from the healthy leg, was significantly higher in SF with respect to NF in both subjects (*P* < 0.05) (Fig. 3B). In subject 1, the increase was of approximately 35%. In subject 2, the increase was subtler (about 3%). In subject 1, we also found a slight increase in the impulse of the vGRF [defined as the integral of the vGRF (table S2) (*41*)], corresponding to an increase of about 10%. In the ascending phases of ST, both subjects showed significantly higher loading peaks and push-off peaks measured on the prosthetic side in the SF condition with respect to NF (*P* < 0.05) (Fig. 3C). Moreover, the push-off peaks, measured on the healthy side, were always higher on both legs of subjects 1 and 2 (*P* < 0.05) (Fig. 3D).

**Fig. 4:**
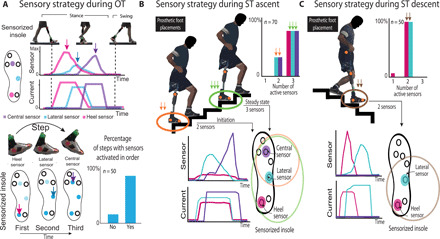
Underlying code of neural stimulation. Stimulation channels activated during OT (**A**), ST ascent (**B**), and ST descent (**C**). Percentage of steps with sensors activated during these tasks according to the steps (OT, *n* = 50; ST ascent, *n* = 70; ST descent, *n* = 50). In OT, the percentage of steps with sensors activated in order (first, heel; second, lateral; and third, central) is shown. On ST ascent, two cases occurred: three (central, lateral, and heel) or two active sensors (central and lateral). On ST descent, only two sensors were activated (lateral and heel). Different temporal order or spatial usage could be a simple, but robust, indicator of intuitively integrated codes for different motor behaviors. (Photo credit: Stanisa Raspopovic, ETH Zurich).

To unveil the underlying code exploited by the subjects, we investigated the activated insole sensors and the neural stimulation channels. This information shows the artificial foot placement strategy of the subjects based on the neural feedback in different tasks. During OT, all the three sensors used to provide tactile information related to heel, lateral met, and central met were always activated during every step. In 95% of the cases, the sensors were activated in order, starting from the heel, then lateral, and lastly central sensor (Fig. 4A). In ST ascent, during the initiation step, the subjects activated two sensors (lateral and frontal sensors) and, during the steady state, all the three sensors together (heel, lateral, and frontal sensors) (Fig. 4B). Considering the entire ascending phase, in 30% of the total steps, two sensors were activated simultaneously and, in 70%, three sensors. Last, in the ST descent, in 98% of the steps, the subjects simultaneously activated two sensors (heel and lateral sensors) (Fig. 4C). Comparing these activations with those observed in the same tasks in NF condition, the subjects adopted different strategies. In OT, the cases in which the sensors were sequentially activated passed from 95% in SF to 82% in NF condition (fig. S6A; Fisher’s exact test, *P* < 0.05, *n* = 50 steps). This result suggests that the subjects did not use the prosthesis as a cane but rather with a more physiological pattern (rolling the foot over the ground), when SF was provided respect to the case without. During the ST descent, the percentage of steps in which the heel and lateral sensors were simultaneously activated decreased to 80% (in 20% of the steps only heel sensor was active), indicating more variability in the foot placement (fig. S6B; Fisher’s exact test, *P* < 0.01). Only in ST ascent, we did not find a significant difference in the sensors activation, but rather a qualitative trend in NF compared with SF condition (in NF condition, 42% of the total steps two sensors were activated simultaneously and, in 58%, three sensors; fig. S6B, Fisher’s exact test, *P* > 0.1). Therefore, different time or spatial order has been used for the different tasks when the SF was provided.

**Fig. 5:**
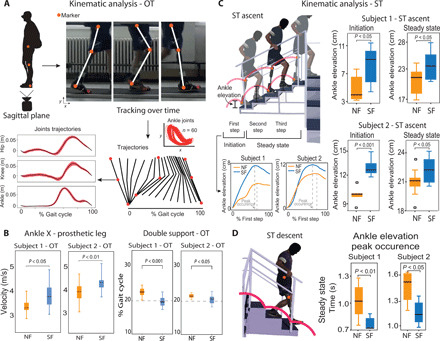
Reshaping of kinematics toward a more physiological gait. (**A**) During OT, main joints markers of the prosthetic leg and of the healthy leg were tracked over time from the sagittal plane. The trajectories, peaks occurrences, and velocities were calculated for each subject. (**B**) For both subjects, horizontal ankle velocities of the prosthetic leg were different in SF with respect to NF (*n* = 24 per condition). Double support as percentage of gait cycle is presented for both subjects in NF and in SF. Dashed line indicates reference normative value. *n* = 46 for subject 1 and *n* = 30 for subject 2 per condition. (**C**) During ST, the leg markers of the prosthetic leg were tracked. Ankle elevation during the initiation (first step) and steady state (second and third steps) are displayed for subject 1 (*n* = 24 per condition) and subject 2 (*n* = 25 per condition). (**D**) The peak occurrences of the ankle elevation and the velocities (*x* and *y*) are shown for each subject during the descending phase of the ST (only for the step with the prosthetic leg, *n* = 24 per condition). All data are shown as box plots for neural sensory feedback (SF) and for without (NF) during OT and ST. *P* values of the Wilcoxon test are shown. (Photo credit: Giacomo Valle, ETH Zurich).

Then, we performed the analysis of the kinematics data (joint trajectories and velocities) (Fig. 5, A and C). In the OT, both subjects had the maximum horizontal velocity of the prosthetic ankle approximately 30 cm/s higher with SF compared to NF condition (*P* < 0.05) (Fig. 5B). When the sensory feedback was provided, the percentage of double limb support during gait decreased for both subjects (Fig. 5B). The percentages of double support (when both feet are in contact with the ground) during SF were more similar to physiological values [around 20% (*42*)].

In subject 1, we also observed this higher speed at the prosthetic knee joint with an increase of horizontal velocity of 30 cm/s (*P* < 0.05) (table S3). Moreover, the ankle elevation and velocity in the *y* direction (direction perpendicular to the ground) slightly increased when the neural feedback was provided (*P* < 0.05) (fig. S5A). In subject 2, we obtained that the maximum horizontal velocity of the ankle and knee in the healthy leg was higher in the SF condition (fig. S5A). In the ankle joint, there was an increase of about 40 cm/s, while in the knee joint, the increase was of about 10 cm/s (*P* < 0.05) (table S4). Subject 1 also presented a higher maximum vertical velocity in the prosthetic knee while walking with neural sensory feedback (*P* < 0.05) (table S3). The increase in both joints was approximately 10 cm/s.

In the ascending phase of ST, the ankle elevation was higher in the SF condition compared to the NF condition (Fig. 5C and movie S2) for both subjects (*P* < 0.05). This result was consistent in the initiation (first step) and during the steady state (second and third steps). Moreover, by analyzing the time occurrences of the peaks of the ankle joint trajectories and velocities for each step (defined as shown in Fig. 5C), we observed a lower value in the second and third steps, when the neural feedback was provided with respect to NF condition (*P* < 0.05) (fig. S5A). Same results were obtained for both subjects also in the descending phase of ST (*P* < 0.05) (Fig. 5D and fig. S5C). This reduction in the time occurrences showed the increment in ankle joint velocity in the SF condition.

**Fig. 6:**
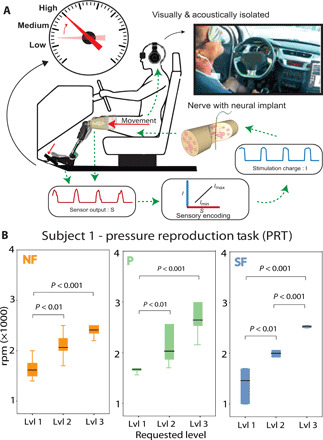
Proof-of-concept scenario for the future neuro-leg prostheses. (**A**) During the fine pressure reproduction task (PRT), the participant was blindfolded and acoustically shielded. When the subject pressed on the pedal to reproduce the level of pressure requested, the simultaneous readout from insole sensors were feed back to the user through intraneural stimulation using TIMEs. The requested level was recorded in revolutions per minute (rpm). (**B**) rpm measured during PRT according to the level requested. Data are shown as box plots with neural sensory feedback (SF), with position feedback (P), and without neural sensory feedback (NF) (*n* = 48). In plots: 1, low; 2, medium; 3, high force levels. *P* values of the Kruskal-Wallis test with Tukey Kramer for multigroup correction are shown. (Photo credit: Giacomo Valle, ETH Zurich).

Exploiting sensory feedback for future neuro-robotic scenarios, subject 1 performed a task in which he exerted different levels of pressure on a car accelerator pedal with the prosthetic leg (Fig. 6A). The task measured the ability to reproduce fine pressure levels with the prosthetic leg [pressure reproduction task (PRT)]. The subject was asked, in a random order, to apply three levels of pressure on the pedal (low, medium, and high), relying only on the feedback from the prosthesis (while blindfolded and acoustically insulated). In particular, the subjects had to apply the pressure at the required level, maintain it for approximately 2 s, and then release.

The overall performance was 95.3 and 67.2%, respectively for SF and NF. Subject 1 was able to consistently modulate the pressure force at the three different levels only in the SF condition (Fig. 6B). In NF, levels 2 and 3 were not statistically different, indicating that the subject was able to reproduce only two different levels of pressure. On the other hand, in the SF condition, all the three force levels were statistically different (*P* < 0.01). We tested also a condition providing only position feedback (P). In this condition, only two levels were statistically different, as for NF (only levels 1 and 3), with a precision of 80.5% (Fig. 6B).

## DISCUSSION

Lower-limb amputees suffer lack of sensory feedback that affects the correct sensorimotor integration (*43*) between the central nervous system and the prosthetic limbs. The benefits of restoring sensory information in an artificial leg using intraneural stimulation were recently reported (*25*, *26*). The results of our detailed analysis demonstrated the impact of intraneural sensory restoration on meaningful gait parameters (i.e., from which walking improvements can be spotted) and on sensorimotor strategies in transfemoral amputees. We hypothesize that these schemes, promoted by the connection between the prosthesis and the nervous system, reflect in improved mobility during motor tasks. The improvement of the mobility was observed in tasks of different difficulties.

In particular in OT, we found that with sensory feedback the subjects have increased mobility with a gait speed being respectively 10 and 5% higher than in the NF condition in subjects 1 and 2. We found that in both subjects, different motor strategies may have led to this increase in speed: (i) increase in walking cadence and (ii) increase of stride length.s

Although generally in healthy individuals a higher gait speed is accompanied by a higher cadence (*44*), this is not the case in transfemoral amputees (*41*). Here, the stepping frequency (cadence) of both subjects was higher in the SF condition. In both subjects, we noted no significant difference in swing time in both legs between feedback conditions. On the other hand, we found that in both subjects, in both prosthetic and healthy legs, the stance time was significantly reduced in the condition with feedback. With a change of stance time and no change in swing time, we found that the time was being saved during the double-support phase (Fig. 5). A probable reason for this is a faster shifting of load from the healthy to the prosthetic leg and vice versa (*45*). Thus, in our case, we hypothesize that the subjects switch load from one leg to the other faster with feedback than without. This is likely due to higher confidence (Fig. 1F) in the prosthetic leg with sensory feedback (*25*), which we measured quantitatively here (Fig. 3). The amputee is able to instantly sense the position of his leg with regard to the ground, which allows him to transition faster from heel strike to loading his prosthetic leg.

The second mechanism for achieving higher walking speed in SF was the increase of stride length. Both subjects showed an increase of around 6% in stride length with sensory feedback as compared to with no feedback. A longer stride length is a preferred strategy for achieving higher gait speeds in transfemoral amputees (*46*, *47*). A higher stride is a sign of higher gait confidence (Fig. 1F) (*40*). If the leg in swing phase is not set down properly, then it is much harder to maintain balance when the center of mass has been shifted forward (a longer stride can then be seen as a greater risk). To find the mechanism for achieving a longer stride with constant swing time (i.e., the foot traveled a higher distance with the same time), we investigated the knee and ankle joint velocities of both legs. We found that the higher stride was accomplished by a faster swing of the prosthetic leg (Fig. 2). We found a higher maximum horizontal velocity in both the ankle and knee joint of the prosthesis (Fig. 5A). This was accompanied by a higher vertical velocity in both joints (fig. S5).

When the sensory feedback was provided, the gait speeds of the two transfemoral amputees were increased also during stairs ascent and descent (ST), even in more percentage than during the OT. The mechanism that the subjects probably used to walk faster when neural feedback was provided was not only the reduction of their stance time but also the reduction of the swing time. The reduction in swing time means that during the SF condition, the subjects were quicker in correctly placing their prosthetic foot onto the stair, which is a considerably longer process (Fig. 2E) when they have no sensory feedback. When the subjects did not receive any sensory feedback, they could only rely on their visual guidance during the swing phase to see where they were stepping into and to avoid bumping their prosthetic foot against the stair. The visual system is not suitable for extracting salient tactile information from the scene (i.e., applied force or contact events) (*48*).

Considering the results of the forces, in OT, we observed that in both subjects, there was a significant increase in the healthy leg’s loading peak in the SF condition. This is expected with higher gait speeds and can be explained by higher deceleration of the leg upon impact with the ground. These findings are in accordance with literature and can be explained by higher accelerations at higher walking speeds (*25*, *41*).

In addition, in ST, we obtained higher forces (vGRFs) on both the healthy and the prosthetic leg in the SF condition because the subjects reached higher velocities (*46*). Thus, feedback restoration boosts the amputees’ confidence (*25*) by making them aware of their prosthetic limb (Fig. 3). Here, we made quantitative measure that confirms the subjective feeling (Fig. 1, F and G). This, in turn, helps them walk faster and trust more their prosthetic limb. Higher velocity during SF condition is associated with higher accelerations, which result in higher applied forces.

By studying the behavior of the neural stimulation during OT, all the foot sensors were active in the stance phase of every step. Moreover, the three sensors were activated in 95% of the cases in an orderly manner (Fig. 4A). In this way, the subjects were able to exploit the neural sensory feedback as an indicator of meaningful spatial (which part of the foot is in contact with the ground), loading (if they are applying low or high force on the prosthesis), and temporal events (i.e., sensation of heel contact, full weight, and push-off) during gait.

Contrary during the ST condition, we found that the spatial information of the tactile feedback provided to the subjects was informative of the correct foot placement on the stair. During the ascending phase, because the entire prosthetic foot was placed on the stair, all the foot sole sensors were often active (guaranteeing the perception of central, lateral met, and heel) (Fig. 4B). Instead on the descending phase, because only half of the foot was placed on the stair to allow the mechanical flexion of the prosthetic knee, only two sensors (eliciting sensations of lateral met and heel) were active (Fig. 4C). In this way, the subjects exploited the sensory information to quickly understand whether the current step would be well placed on the stair and stable, making their speed and prosthesis confidence higher.

In both OT and ST, different temporal or spatial sensor activations have been exploited by the subjects when the SF was provided compared to NF condition (fig. S6). This is a clear indication that previously unknown sensorimotor strategies are being promoted when the sensory flow of information has been restored thanks to this neuroprosthetic device.

In both tasks, the position of the prosthesis in the space was provided by the knee stimulation. The information of the knee flexion/extension was crucial during the swing phase (Fig. 1, B and C), in particular during the ST (Fig. 2E). These evidences showed that even a simple prosthesis sensorization with only three foot sensors and a knee encoder is enough to substantially improve motor performance.

Analyzing the kinematics for ST, without sensory feedback, the subjects have to rely mainly on visual feedback that is not suitable to extract tactile and proprioceptive cues from the environment (*48*). For this reason, in ST ascent, the control of the prosthetic leg is quite difficult for the subjects. When the sensory feedback is provided, the subjects felt the flexion of the knee (position feedback), and they were more aware of the position of the artificial limb in the space. Considering that the aim of the task was to execute the steps at the highest comfortable velocity of walking, their possible adopted intuitive strategy could be based on the combination of spatial and temporal cues. In particular, the subjects received the spatial (position of the prosthetic leg in the space), the temporal (maximal swing), and intensity (flexion of the knee) information to improve their mobility. Our hypothesis is that the subjects were searching rapidly to reach with certainty (therefore at higher ankle point) this combined information while ascending the stairs resulting in a higher speed. We also observed a time reduction of the peak occurrences of the ankle elevations and velocities in the steady state (second and third steps) in both the ascending and descending phases (fig. S5, B and C). When the neural sensory feedback was provided in both the ST phases, the subjects were able to elevate more and faster the ankle to obtain such gait speed increase. Similar results were observed in the stair climbing with the epidural stimulation (*49*).

Simulating a scenario of future use of the neuro-integrated prosthesis, one subject exploited the system to regulate the pressure applied on a gas pedal (Fig. 6). In this experiment (PRT), the subject mastered a precise sensing of the pressure elicited by his voluntary movement, using tactile and position feedback provided by intraneural stimulation. This level of precise pressure reproduction is not reachable with currently available prostheses because of the lack of sensory feedback provided to the user. The participant intuitively and precisely integrated the information provided by the restored feedback in his control loop to accomplish this task. We found that while in the NF condition, subject 1 was only able to reproduce two distinct pressure levels, with neural sensory feedback, he could reproduce three different levels. Because of the feedback from the knee and the one from the foot, he was able to reach much higher accuracy.

Confidence and mobility resulted to be among the clearest and simplest parameters showing the impact of sensory feedback on gait. Therefore, we suggested to consider these important features being the global evidences of a better sensorimotor strategy adopted by the subject. This could be of great importance in the design and the evaluation of the benefits of new somatosensory neuroprostheses.

The amputees that participated in these experiments are two proficient prosthetic users [both had a K4 level (*25*)]; therefore, the significance of the results obtained in this gait analysis could potentially be underestimated, because of their diminished space for improvement. The significance of the effect of sensory feedback in transfemoral amputees during stairs ambulation could possibly be increased by analyzing less experienced prosthetic users. Because the results have shown that the system is more useful in a more demanding task (ST), our hypothesis is that this approach could reduce the rehabilitation time after amputation necessary for a complete recovery [e.g., improving the learning curve by decreasing the training time for new prosthesis adoption from 6 to 9 months (*50*)].

Our findings unveiled that spatially and temporally matched restored sensations help in daily life. More recently, innovative sensory encoding strategies of intraneural stimulation, based on computational (*51*, *52*) and biomimetic models (*53*), have been presented as very promising both for functional and cognitive aspects for bidirectional hand prostheses (*54*, *55*). It would be interesting to test these neurally-inspired sensory encodings also in sensorized prosthetic legs, evaluating the effect of using biomimetic sensory feedback on the mobility and on the biomechanics of gait.

There are some limitations to the present study. More experiments with a larger cohort of patients together with a more accurate and advanced acquisition and recording system are necessary to fully understand all the aspects related to the biomechanics generated by the neural sensory feedback adoption. Moreover, more tasks of daily living have to be tested to evaluate the benefits of the sensory feedback restoration in a home-use adoption. In particular, the OT has to be tested also without visual restriction to quantify and confirm the improvements in a more common situation.

In conclusion toward future wide-spread use of neuro-robotic prostheses (*56*), our findings showed how to improve and to promote effective sensorimotor strategies guaranteeing increased mobility in leg amputees. This approach allowed two transfemoral amputees to improve their gait speed and confidence during possible motor tasks of daily life. When the sensory information, delivered via intraneural stimulation, was provided, the spatiotemporal, balance, and kinematics gait parameters were improved in both subjects. Last, we demonstrated that sensory feedback guarantees a finer pressure reproduction with the prosthetic leg. These results demonstrate that neural sensory information exploited in a real-time sensorized leg prosthesis changes the sensorimotor strategies of gait, leading to an improved prosthesis operation in possible motor tasks of daily living. The functional results pave the way toward more sophisticated bionic legs (*57*), conveying rich and multimodal sensations.

## MATERIALS AND METHODS

### Patient recruitment, experiment logistics, and surgical procedure

Two right transfemoral amputees [level K4 (*58*)] were included in the study. The first subject (subject 1) was a 49-year-old male, who had a traumatic transfemoral amputation of the distal two-thirds of the right leg 3 years before the enrollment in the trial. The second subject (subject 2) was a 35-year-old male, with a traumatic transfemoral amputation of the distal two-thirds of the right leg, which occurred 12 years before the study. Both of them were active users of passive prosthetic devices (Ottobock 3R80) (table S1).

Ethical approval was obtained from the institutional ethics committees of the Clinical Center of Serbia, Belgrade, Serbia, where the surgery was performed. All the subjects read and signed the informed consent. During the entire duration of our study, all experiments were conducted in accordance with relevant European Union guidelines and regulations.

Four TIMEs (*31*) (14 active sites each) were implanted in the tibial branch of the sciatic nerve of each subject. The surgical approach used to implant TIMEs has been extensively reported elsewhere (*25*). Briefly, during general anesthesia, through a skin incision over the sulcus between the biceps femoris and semitendinosus muscles, the tibial nerve was exposed to implant four TIMEs. The microelectrodes and a segment of their cables were drawn through four small skin incisions 3 to 5 cm higher than the pelvis ilium. The cable segments were externalized (and secured with sutures) to be available for the transcutaneous connection with a neural stimulator. After 90 days, the microelectrodes were removed under an operating microscope in accordance with the protocol and the obtained permissions.

This study was performed within a larger set of experimental protocols aiming at assessing the impact of the restoration of sensory feedback via neural implants in leg amputees during a 3-month clinical trial. The subjects presented in this study are the same participants from (*25*, *26*); however, the data shown in this paper are totally new. Here, we explore the mechanisms of how the restoration of artificial sensations in the sensorimotor loop promotes effective motor strategies (measured through performances, kinematics, and dynamics) and which is the plausible way in which users exploit these artificial precepts. The data reported in this manuscript were obtained over a period of several days in two leg amputees during the second and third month of trial (i.e., after the electrode implantation). Tasks were randomized. Because of limited time availability, subject 2 decided not to participate in the fine PRT.

### Sensorized bionic leg

The system used in this study was the same used by Petrini *et al.* (*25*, *26*). In particular, the sensorized bionic leg was composed of (i) a microprocessor-controlled prosthesis (RHEO KNEE XC, Pro-Flex XC foot and transfemoral flexible brim socket fitted to an Iceross Seal-In X5 TF silicon liner) with an integrated 14-bit knee encoder; (ii) a sensorized insole, purposely developed for this neuroprosthesis (SensArs Neuroprosthetics), placed under the prosthetic foot. The insole constituted of a substrate of fabric, on which seven pressure sensors were positioned. The sensors had a resolution of 0.05 kg and a maximum measurable weight of 100 kg. The acquisition and amplification system of the sensorized insole had a sampling frequency of 75 Hz and a Bluetooth module. (iii) An external controller (implemented on Raspberry Pi 3, Raspberry Pi Foundation) was connected, via Serial Peripheral Interface (SPI) communications, to the external neural stimulator (see below) and communicated via Bluetooth with both the sensorized insole and the RHEO KNEE XC. This portable processor regulated the acquisition and recording of sensor readouts and the sensory encoding algorithm, transducing it into stimulation parameters needed for driving the neural stimulator. (iv) A real-time controllable neural stimulator [STIMEP, Axonic, and University of Montpellier–Laboratorie d’Informatique, de Robotique et de Microelectronique de Montpellier (LIRMM) (*59*)] whose four channels connected to four active sites of the electrode implanted in tibial nerve are driven by the readouts of three insole sensors and the knee encoder. Just the insole size was adapted to best fit with the dimension of the prosthetic foot.

### Intraneural stimulation for tactile and position feedback

Each of the four TIMEs implanted in both amputees was constituted by 14 active sites and two ground electrodes. For each subject, 56 electrode channels were accessible for stimulating. During the characterization procedure, the stimulation parameters (i.e., amplitude and pulse width of the stimulation train), for each electrode and active site, were obtained. The scope of this procedure was to determine the relationships between stimulation parameters and the quality, location, and intensity of the electrically evoked sensation, as described by Petrini *et al.* (*26*). In brief, the injected charge was linearly increased at a fixed frequency [50 Hz (*26*)] and pulse width by modulating the amplitude of the stimulation for each electrode channel. In case the stimulation range was too small for the chosen pulse width and the maximum injectable amplitude, the pulse width was increased, and the same procedure was repeated. In the moment that the subject reported to perceive any electrically evoked sensation, the minimum charge (i.e., perceptual threshold) was registered. The maximum charge was collected to avoid that the sensation became painful or uncomfortable for the subject. This was repeated five times per channel and then averaged. Perceptual threshold and maximum charge were obtained for every electrode channel and have been used to choose the stimulation range. For each active site, the maximum injected charge was always below the TIME chemical safety limit of 120 nC (fig. S1b) (*60*).

The force measured by the insole sensors was conveyed into neural stimulation using a linear amplitude modulation (*61*) implemented to associate the perceived intensity with measured pressure. The stimulation parameters were selected to exploit the whole dynamic range of the sensations for each participant. The number of functional active sites of TIMEs, the ranges of neural stimulation, and whether the active sites/charge changed during the experiment are reported by Petrini *et al.* (*26*). The stimulation parameters and sensations exploited during the motor tasks are reported in fig. S1. Last, one insole sensor was placed in the forefoot area related to a sensation in the phantom forefoot, one to the midfoot, and one to the hindfoot (Fig. 1A). The knee encoder controlled an active site eliciting a muscle contraction (for subject 1 and pressure on the muscle for subject 2) of the gastrocnemius (Fig. 1A). To find the most reliable electrode channels and sensations to use during the experiments, the characterization procedure was performed every week.

The tactile feedback was homologous (pressure sensations) and somatotopic (referred under the phantom foot). The position feedback for the knee extension/flexion resulted to be both quasi-homologous (perception of leg flexion/extension) and quasi-somatotopic (referred on the phantom leg). The combination of “muscle contraction” and prosthesis motion was perceived by the subjects as phantom knee specific angle position and its variation (i.e., flexion/extension of the knee) as reported in (*26*).

### Experimental setup

The experimental setup for the gait parameter acquisition was designed to collect spatiotemporal, balance, and kinematics data while the subjects were performing motor tasks. In particular, two sensorized insoles (SensArs Neuroprosthetics) were placed under the prosthetic foot and the healthy foot. Each insole contained seven pressure sensors and an acquisition system able to record pressure (balance) information on both sides during the gait. In addition to the sensor data, the subjects were outfitted with tracking markers on their leg joints. Two cameras (HDR-CX240E Handycam, Sony) were used for recording videos from two planes of motion in all the tasks (the sagittal and frontal planes). Even though three-dimensional (3D) motion recording is considered as the “gold standard” for analyzing kinematics, using 2D cameras, when properly placed and calibrated, results in reliable data with neglected measurement errors for this purpose (*62*–*65*). Cameras were fixed with a tripod and placed on a 3-m distance from the obstacle setup to be able to include whole space of motion and that all segments appear to be in a single plane (*xy*).

The markers were positioned on the prosthetic and healthy legs of the subjects. In the frontal plane, hip markers were positioned on the Iliac crest; knee markers were positioned on the patella (kneecap), and ankle markers were positioned at the meeting of the talus and tibia (Fig. 5A). In the sagittal plane, hip markers were positioned at the femur head, knee markers were placed at the junction of the femur and tibia, and the ankle markers were positioned at the bottom of the lateral malleolus. In addition, two markers were placed over the outermost toe of the subjects. Lateral hip, lateral ankle, and knee position data from both legs were offline extracted for the OT and ST.

Data extraction was done in Kinovea (freeware). Markers were tracked to extract joint trajectories during the gait tasks. The coordinate system was calibrated by entering the actual height of the stairs and length of the walking support (*65*). Considering that the camera were perpendicular to the movement plane and the aspect ratio of the camera’s pixels was known (4/3 with a rate of 25 frames per second), a defined and fixed distance was used to scale the pixel information into metric information. As one foot was always positioned closer to the camera than the other one, the calibration was performed separately for the right and left foot. Then, using Kinovea tracking software, the markers could be tracked to extract joint trajectories during the gait tasks.

### Over-ground walking task

In this task, subjects were asked to walk on a flat runway of a length of 4 m (Figs. 1B and 2A) during 2-min sessions. Between sessions, the subjects were allowed to rest for a suitable period of time (approximately 10 min) to avoid fatigue bias. The subjects were asked to walk at their preferred pace and were not given any indications to adjust their gait. The runway was equipped with handlebars that the subjects were asked to use sparingly. To avoid the subject receiving visual feedback on the position of their prosthetic leg, they wore blinders that restricted their vision in such a way that they were unable to see below their waist. Walking sessions were performed in two distinct conditions: (i) no feedback (NF): in this condition, the subjects did not receive any sensory feedback; and (ii) neural sensory feedback (SF): in this condition, the subjects received feedback from both the sensorized insole and the prosthetic knee encoder (see the “Intraneural stimulation for tactile and position feedback” section). All the stimulation conditions were randomly presented to the volunteers. During the task, one camera was placed in the sagittal plane allowing to track the joint trajectories (right or left depending on walking direction). Another camera was paced in the frontal plane; joints could be tracked when the subject was walking toward the camera.

In our study, speed was calculated as the time it took for a subject’s center of mass to travel between vertical bars at opposing ends of the runway, separated by 4 m. Time values were extracted manually from the sagittal plane videos. The speed was calculated in meters per second. Cadence is the stepping frequency, expressed in steps per minute. In our case, cadence was extracted manually from the sagittal plane video by timing the interval between successive heel strikes of opposing feet. Stride length is the distance between two successive heel strikes of the same leg. It was extracted by tracking the ankle marker in the sagittal plane with Kinovea. In this study, stance and swing time were extracted from the videos by noting the times of heel strike and toe-off of each leg. Another related measure is double-support time, which was computed from the gait cycle. This is the percentage of the gait cycle in which both feet are in contact with the ground. Both subjects performed the task 30 times per condition (randomized).

The patients’ walking dynamics was studied through vGRFs. These were obtained from the insole output by summing the values of all seven sensors. Before proceeding to use the sensors, it was necessary to assess the quality of each sensor to remove any defective elements. Although the shape of this ground reaction force is similar to what could be seen using force plates, its magnitude is considerably lower as the contact area between foot and ground is much larger than the surface covered by the pressure sensors. This is consistent with previous works when the vGRF is calculated using sensorized insoles (*66*, *67*). The estimation of the force was repeatable, and the same insoles were used in all the (randomized) tasks and conditions; therefore, we made a comparison between the sensory conditions using the same setup. We used the vGRF (N) to calculate the loading peaks and push-off peaks (and also the impulse) (Fig. 3A) (*41*, *68*).

In the kinematics assessment, we analyzed the joint trajectories and velocities extracted via the camera and marker setup (Fig. 5A). The parameters that we analyzed for each joint were (i) *y*-axis trajectory; (ii) *x*- and *y-*axis velocities—derived parameters from the respective trajectories; (iii) the *x*-axis trajectory.

The methods and parameters explained in this section are applicable to both prosthetic and healthy legs.

### Stairs task

During the stairs test, the subjects were asked to go through a course of stairs in sessions of 30 s per 24 times per condition. The setup was configured as an angular staircase endowed with six steps with a height of 10 cm and a depth of 28 cm on one side and with four steps with a height of 15 cm and a depth of 27.5 cm on the other. Subjects were asked to walk clockwise climbing up the six steps and going down the four steps (Figs. 1C and 2D). Walking sessions were performed in two distinct conditions: (i) no feedback (NF): in this condition, the subjects did not receive any sensory feedback; and (ii) neural sensory feedback (SF): in this condition, the subjects received feedback from both the sensorized insole and the prosthetic knee encoder (see the “Intraneural stimulation for tactile and position feedback” section). All the stimulation conditions were randomly presented to the volunteers. During the task, one camera was placed in the sagittal plane with respect to the ascending part of the stairs, allowing to track the joint trajectories during the ascent phase (only the prosthesis was visible). Another camera was placed in the sagittal plane with respect to the descending part of the stairs (only the prosthesis was visible), allowing to track the joints when the subject was descending the stairs.

The gait speed for this task was reported in terms of number of laps. A lap is intended as going up and down the stairs and reaching the starting position again. A higher number of completed laps is indicative of a higher speed and vice versa. All the spatiotemporal parameters (stance and swing time) were extracted from the video recordings. Stance time is considered the time from heel strike to toe-off, whereas swing time is considered the time from toe-off to the next heel strike.

An example of what the sensor outputs look like and the way in which the vGRF is computed is given in Fig. 3A. The example is about the case of OT; however, the method for ST is analogous. From the vGRF, we calculated (i) the loading peak (the first peak that appears in the vGRF) that represents the force that the subject uses to load his leg and (ii) the push-off (propulsion) peak that is the force that the subject uses during the late phase of stance to push forward (Fig. 3A).

Using the Kinovea software and the markers that the patients worn during the experiment, we could track these joints (Fig. 5C). The parameters that we analyzed for each joint were (i) *y*-axis trajectory; (ii) *x*- and y-axis velocities—derived parameters from the respective trajectories; (iii) the *x*-axis trajectory is not considered during the analysis. The reason for this is the fact that the *x*-axis trajectory is restricted by the stair dimensions, as opposed to the *y*-axis trajectory where the patient can freely lift his leg in the vertical position. We calculated the peak occurrences of the ankle elevation and velocities in the ascending and descending phases for both feedback conditions.

The methods and parameters explained in this section are applicable to both ascent and descent.

During ascent, the analysis is reported separately between the first step and the second and third steps. This method is in accordance with (*69*). The initiation and termination period, i.e., the first stair/step ascent and the last stair/ascent, should be differentiated from the steady-state stair ambulation. This is because the step from the lower platform to the first step does not cover the same trajectory as the step from the first step to the third one or from the third step to the fifth one. For the descent phase, we did not consider the initiation and termination phase of the stair descent. We only considered the steady-state stair descent (only one step). The reason for this is, in part, due to the different descending strategies that the patients use. Subject 1 started descending with his healthy leg. This implies that the prosthetic leg (the one that we have analyzed kinematics for) was used to descend from the upper platform (first step) to the third step. The next step that subject 1 did was from the third step to the lower platform, but we chose to ignore this trajectory because toward the end of the descent phase, the patients took a turn to get to the ascending part of the stairs, and this might negatively affect the trajectory analysis. Subject 2, on the other hand, started the descent with his prosthetic leg. This is the initiation phase and we did not do the analysis for this step, because as it was explained, subject 1 did not perform this step with his prosthetic leg. The next step of subject 2 was from the second step to the fourth step, and this is part of the steady-state descent that we considered. The last step for subject 2 was from the fourth step to the lower platform, and this was the termination phase; however, for the same reasons as subject 1, we chose not to consider this as part of the trajectory analysis.

### Self-reported confidence assessment

At the end of each session of OT and ST, participants were asked to assess their self-confidence while performing the motor task, using a visual analog scale (from 0 to 10). The data were acquired in SF and NF conditions in both subjects.

### Fine PRT

In this experiment, the subjects’ ability to produce different pressure levels with their prosthetic leg was assessed (Fig. 6A). This experiment included only subject 1. The subject was instructed to rely on the sensory feedback information to reproduce three different levels of pressure. The experiment was performed in three conditions: (i) no feedback (NF): subject did not receive any sensory feedback; (ii) position feedback (P): subject exploited the position feedback only; (iii) sensory feedback (SF): subject was provided by tactile and position information from both the knee encoder and the insole sensors. The subject was seated in the driver position of a car and asked to produce three different pressure levels corresponding to three different revolutions per minute (rpm) levels (low, medium, and high) by pressing on the accelerator pedal with his prosthetic limb. He was blindfolded and acoustically insulated with earplugs playing loud music. This was done to ensure that he would not depend on auditory or visual feedback during the task. The experimenter would request a pressure level by touching the arm of the subject a number of times corresponding to the force level required (i.e., one touch for level 1 and two touches for level 2), the subject would then press on the gas pedal of the vehicle and attempt to produce an rpm level in correspondence with the requested force level. To press on the pedal, because the prosthetic ankle was blocked (as per construction) and the knee was instead free to be flexed, the amputee shifted slightly his stump forward (Fig. 6A), inducing a composed effect of a higher knee angle and a higher pressure on the foot. Once confident in his response, the subject would verbally confirm the force level that he was producing. The experiment was filmed to have a record of the force level requested and the rpm level given in response. Ninety trials (30 × 3 levels) were performed in each feedback condition.

### Statistics and data analysis

All data were exported and processed offline in Python (3.7.3, the Python Software Foundation) and MATLAB (R2016a, the MathWorks, Natick, USA). All data were reported as mean values ± SD (unless elsewise indicated). The normality of data distributions was verified. In case of Gaussian distribution, two-tailed analysis of variance (ANOVA) test was applied. Elsewise, we performed the Wilcoxon rank sum test. Fisher’s exact test (*P*) was used to compare the percentage of sensor activations (fig. S6) in OT and ST ascent and descent. Post hoc correction was executed in case of multiple groups of data. Significance levels were 0.05 unless differently reported in the figures’ captions. In the captions of the figures, we reported the used statistical tests for each analysis and its result, along with the numerosity of the distributions. Details about the number of repetitions (*n*) and *P* values for each experiment are reported in the corresponding figure legends.

## SUPPLEMENTARY MATERIALS

Supplementary material for this article is available at http://advances.sciencemag.org/cgi/content/full/7/17/eabd8354/DC1
